# Influence of Tetrabromobisphenol-A on the Fate and Behavior of Zinc Oxide Nanoparticles Affected by Salts, Humic Acid, and Bovine Serum Albumin in Water Systems

**DOI:** 10.3390/toxics13030148

**Published:** 2025-02-21

**Authors:** Anwar Ul Haq Khan, Yanju Liu, Ravi Naidu, Cheng Fang, Ho Kyong Shon

**Affiliations:** 1 Global Centre for Environmental Remediation (GCER), College of Engineering Science and Environment, The University of Newcastle, Callaghan, NSW 2308, Australia; anwar.khan@uon.edu.au (A.U.H.K.); ravi.naidu@newcastle.edu.au (R.N.); cheng.fang@newcastle.edu.au (C.F.); 2 crc for Contamination Assessment and Remediation of the Environment (crcCARE), ATC Building, The University of Newcastle, Callaghan, NSW 2308, Australia; 3 School of Civil and Environmental Engineering, University of Technology Sydney (UTS), City Campus, Broadway, NSW 2007, Australia; hokyong.shon-1@uts.edu.au

**Keywords:** zinc oxide nanoparticles, tetrabromobisphenol-A, salts, adsorption, zeta potential, aggregation

## Abstract

The environmental release of zinc oxide nanoparticles (ZnO-NPs) may have consequences for ecosystems. The behavior and environmental effects of ZnO-NPs could change due to their interactions with other existing substances. This research explored how the presence of coexisting organic pollutants (like tetrabromobisphenol-A (TBBPA)), electrolytes (such as NaCl and CaCl_2_), natural organic materials (including humic acid (HA)), and bovine serum albumin (BSA) in simulated water affected the behavior of ZnO-NPs. Various characterization techniques were used to analyze the size, shape, purity, crystallinity, and surface charge of ZnO-NPs following interactions (after one day, one week, two weeks, and three weeks) at pH 7. The findings demonstrated changes in both the size and zeta potential of the ZnO-NPs in isolation and when TBBPA and electrolytes were included in the suspension. The size and surface charge exhibited different variations across fixed concentrations (5 mM) of various electrolytes. HA and BSA contributed to the dispersion of ZnO-NPs by affecting the zeta potential. These dispersion effects were also observed in the presence of TBBPA and salts, attributed to their substantial aliphatic carbon content and complex structures. Potential interaction forces that could explain the adsorption of TBBPA include cation bridging, hydrophobic interactions, hydrogen bonding, electrostatic interactions, and van der Waals forces. The co-occurrence of organic pollutants (TBBPA) and natural organic compounds (HA and BSA) can alter the surface properties and behavior of ZnO-NPs in natural and seawater, aiding in the understanding of the fate and impact of engineered nanoparticles (such as ZnO-NPs) in the environment.

## 1. Introduction

Zinc oxide nanoparticles (ZnO-NPs), a type of inorganic mineral filter, are widely incorporated into various consumer products, such as cosmetics, paints, electronics, and textiles. Their popularity stems from their ability to effectively absorb and reflect ultraviolet (UV) radiation, providing protection and enhancing product durability [1,2,3]. ZnO-NPs are commonly produced inorganic substances, having around 10 million tons of total worldwide output [4,5]. ZnO-NPs rank as the third most widely produced metal-based ENPs worldwide, following silicon dioxide (SiO_2_) and titanium dioxide (TiO_2_), with annual production estimates ranging from 550 to 33,400 tons. Their release into water, soil, and sediments raises concerns about potential environmental risks, as their nanoscale size, high surface-area-to-volume ratio, and toxic properties can negatively impact various organisms, including plants, animals, microbes, and humans [6,7,8]. Adverse effects on marine life, such as sea urchins, have been documented [9], and daphnia [10], mammals [11], earthworms [12], marine diatoms (*Thalassiosira pseudonana*) [13], and plants [14] have also been reported. Likewise, the accumulation and harmful effects of ZnO-NPs from sunscreens in seawater have been documented [15]. Studies show that exposure to these nanoparticles can lead to growth inhibition, DNA damage, and oxidative stress in marine algae [15].

Various modeling studies based on material flow suggest that ZnO-NPs are released into surface water at concentrations ranging from 0.008 to 0.055 µg/L, while wastewater treatment plant effluents contain between 0.34 and 1.42 µg/L [16,17]. Estimated concentrations of ZnO-NPs in different natural environments have also been reported, including sediment (1.8–5.7 µg/kg/y), soil (6.8–22.3 µg/kg/y), and sludge (136–647 µg/kg/y) [18]. In the United States, wastewater treatment influent has been found to contain ZnO-NPs at levels between 20 and 212 µg/L [19]. Additionally, studies have detected the release of engineered nanoparticles (ENPs), including ZnO-NPs, from sunscreens into swimming pool water [20]. It is estimated that around 10–25% of manufactured ZnO-NPs may enter the environment and accumulate in freshwater systems [21]. The release of ZnO-NPs up to 0.05–10 µg/L (estimated based on model studies) in surface waters in the United States has been investigated [22].

The physical, chemical, and eco-toxicological behaviors of ZnO-NPs are critical for risk assessment upon their release into natural environments (e.g., recreational and swimming waters, wastewater and seawater bodies). Environmental factors, such as electrolytes, pH, organic and inorganic compounds, polymers, light, and heat, can significantly influence the behavior and toxicity of ZnO-NPs [23,24,25]. These interactions can have a substantial impact on their fate and behavior. The presence of proteins, such as BSA, humic substances, ultraviolet radiation, and salinity, can affect their interaction mechanisms [25,26,27]. For instance, organic pollutants, like brominated flame retardants, including polybrominated diphenyl ethers and hexabromocyclododecane, influence the physicochemical properties of ZnO-NPs in aqueous environments under certain conditions [28,29]. However, the potential formation of complex compounds through interactions with metal oxides and polymeric substances has not been accounted for. Studies indicate that upon interacting with HA, ZnO-NPs undergo dispersion and exhibit alterations in surface charge [30]. Electrolytes influence the stability of ZnO-NPs, leading to agglomeration at high salt concentrations due to electrical double-layer compression and reduced energy barriers [31,32,33]. The electrostatic attraction between BSA and the ZnO-NPs surface promotes adsorption, leading to reduced agglomeration and flocculation [34]. The hydrophobic nature of organic pollutants assists them in interacting with and sorbing onto the active sites of ZnO-NPs in aquatic systems. This process is driven by electrostatic and hydrophobic interactions, π–π stacking, van der Waals forces, ligand exchange, hydrogen bonding, and molecular bridging effects [35,36]. 

TBBPA remains one of the most widely used brominated flame retardants. In 2016, around 241,352 tons were produced, primarily in China, the USA, and the Middle East [37]. China produces approximately 180,000 tons of TBBPA annually [37]. TBBPA is primarily used in epoxy resins and polycarbonate, which are found in products such as electronics, furniture, keyboards, and other items [38]. TBBPA has been detected in indoor air and dust [39,40], sediments [41], soils [42], water [43], and sewage sludge, leading to its presence in the food chain [44]. TBBPA levels have been reported in various water bodies worldwide. In the River Skerne, a tributary of the River Tees in England, concentrations ranged from undetectable to 9800 ng/g dw. An unidentified river in the Netherlands had concentrations between 0.1 and 130 ng/g dw [45], while levels in South Korea’s Nakdong River varied from 0.05 to 150 ng/g dw [46]. In eastern and southern China, TBBPA was found at concentrations ranging from below detection limits to 4870 ng/L [47]. Similarly, in the USA, measurements in the Detroit River and other industrial areas revealed concentrations between 600 and 1840 ng/L [45]. In China, TBBPA levels of <0.4 to 259 ng/g dry weight were detected in 52 sludge samples from 30 wastewater treatment plants [48].

The release of TBBPA and its derivatives leads to widespread contamination through multiple pathways, particularly when TBBPA interacts with co-contaminants. The co-presence of TBBPA with other compounds in water may impact the fate and behavior of zinc oxide NPs, but knowledge of the interaction mechanisms involved is currently lacking [25,28,49]. This research highlighted altered behavior of ZnO-NPs when interacting with TBBPA, an organic contaminant, under different environmentally relevant conditions in the water. Specifically, the study evaluated the changes in the physicochemical properties, particle size colloidal stability, and ZnO-NPs’ zeta potential before and after they interacted with TBBPA in simulated aquatic environments containing electrolytes, bovine serum albumin (BSA), and humic acid (HA). The findings are crucial for evaluating the potential exposure to ZnO-NPs and associated contaminants under specific water conditions.

## 2. Materials and Methods

### 2.1. Chemicals

ZnO-NPs and electrolytes were acquired from Sigma-Aldrich Australia (Melbourne), and their properties were detailed in a previous study [28]. Briefly, most nanoparticles were less than 100 nm in diameter, with some particles reaching ≥100 nm due to agglomeration. X-ray diffraction (XRD) analysis confirmed that these samples exhibited a hexagonal wurtzite structure. TBBPA (3,3′,5,5–tetrabromobisphenol-A, 97%, CAS: 79-94-7, C_15_H_12_Br_4_O_2_, MW: 543.87 g/mol, mp: 178–181 °C), HA (humic acid technical, CAS: 53680-50G), and BSA (bovine serum albumin lyophilized powder, ≥96%, CAS: 9048-46-8, water soluble at 40 mg/mL for agarose gel electrophoresis) were also purchased from Sigma-Aldrich Australia and used in the study.

### 2.2. Interaction Between ZnO-NPs and TBBPA

To prepare the stock suspension of ZnO-NPs, 0.1 g of ZnO-NPs was added to 1 L of Milli-Q water and sonicated for 10 min. Different TBBPA concentrations were synthesized in Milli-Q water with the ZnO-NPs suspension (0.1 g/L), specifically, 0, 0.5, 1, 10, 50, 100, 200, and 500 µg/L, as well as 1, 5, and 10 mg/L. The increased levels of TBBPA were utilized to enhance the observable effects of interactions measurable by the zeta sizer and particle size analyzer, aiding in the comprehension of the interaction mechanisms. Detecting such alteration effects at environmentally relevant concentrations of TBBPA is challenging because of the constraints in characterization procedures. NPs underwent analysis through techniques including size assessment, zeta potential measurement, dissolution testing, adsorption analysis, TEM, and infrared, both before and following their association with TBBPA at different time points, specifically after one day, and one, two, and three weeks, to evaluate the alterations in behavior of associating NPs compared to the behavior of the pure ones.

Fixed concentrations (such as 5 mM) of two salts (such as NaCl and CaCl_2_) were utilized to examine their impact on NPs’ stability both independently and in conjunction with TBBPA. The influence of HA was also assessed with and without the presence of 10 and 500 µg/L of TBBPA. To prepare the stock solution, HA was added in 0.1 M NaOH solution. Zinc oxide NPs and HA mixers in a range of concentrations were prepared with or without TBBPA. Additionally, the effects of BSA, another organic compound found in nature, on the behavior of ZnO-NPs were studied regarding any modifications in size, shape, and zeta potential of NPs in relation to TBBPA, electrolytes, HA, and BSA.

The dispersed samples were subjected to centrifugation at 18,407 RCF (relative centrifugal force) for 30 min using an Eppendorf Centrifuge 5424 (Sigma-Aldrich, Taufkirchen, Germany). The supernatants were transferred into new 10 mL centrifuge tubes for subsequent analyses, including dissolved zinc and LC–MS analysis. The quantity of TBBPA that adhered to dissolved NPs was assessed after interactions of one day and two weeks. The amount (mg/g) of TBBPA that was adsorbed ($Q_{t}$) was calculated using the formula: $Q_{t}=\frac{(C_{0}-C_{t})*V}{W}$, where $C_{0}$ and $C_{t}$ denote the concentrations of TBBPA in the aqueous solution (µg/L or mg/L) before and following the sorption experiments, respectively. Here, V (mL or L) indicates the volume of the solution, while W represents the mass (mg or g) of the ZnO-NPs. Sample pH was kept at 7 by employing a buffer made of potassium dihydrogen phosphate.

### 2.3. Characterization Techniques

To study morphological changes, including those in water suspensions and after exposure to TBBPA, HA, BSA, and salts, nanoparticles were deposited on TEM grids for observation. Surface areas of ZnO-NPs were analyzed using a Micromeritics TriStar II system, an X-ray diffraction (XRD) system (Empyrean Malvern Panalytical), and field emission scanning electron microscopy (FE-SEM), as described in previous research [28]. FTIR was utilized to detect interactions (functional groups). ZnO-NPs were isolated from the suspension through high-speed centrifugation before FTIR analysis. Changes in NPs’ charge and size, individually and after exposure to TBBPA, HA, BSA, and electrolytes in Milli-Q water, were examined using a Malvern Panalytical Zetasizer. The dissolved zinc concentration was determined through inductively coupled plasma optical emission spectroscopy (ICP–OES; Agilent, Manchester, UK). Additionally, an Agilent LC–MS system was employed to quantify TBBPA adsorption and its associations.

## 3. Results and Discussion

### 3.1. Interaction Between ZnO-NPs and TBBPA

**Figure 1:**
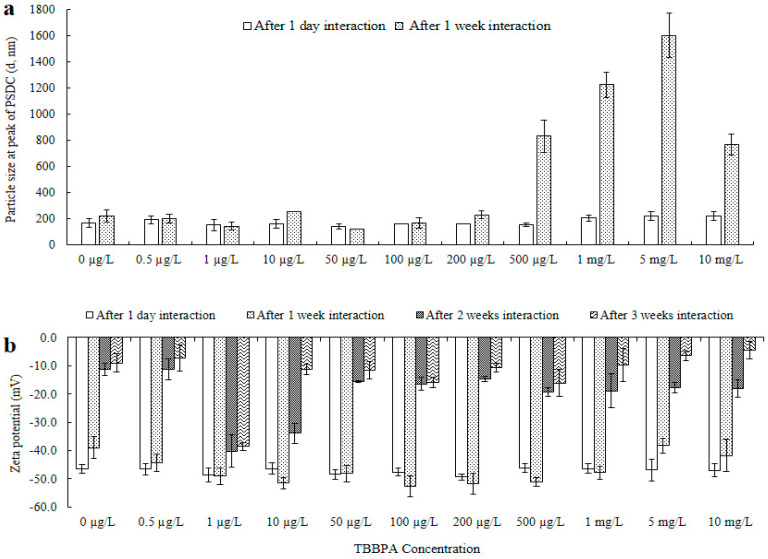
ZnO-NPs’ sizes at the peak of PSDC (**a**) and electrical potential (**b**) after one day and one to three weeks of association with TBBPA. Original particle size at the peak of PSDC was 166 nm for one day and 222.5 nm for one week, and the zeta potential was −46.5 mV for one day, −39.0 mV for one week, −11.3 mV for two weeks, and −9.0 mV for three weeks. Where, “-” represents minus sign in Figure 1b.

**Figure 2:**
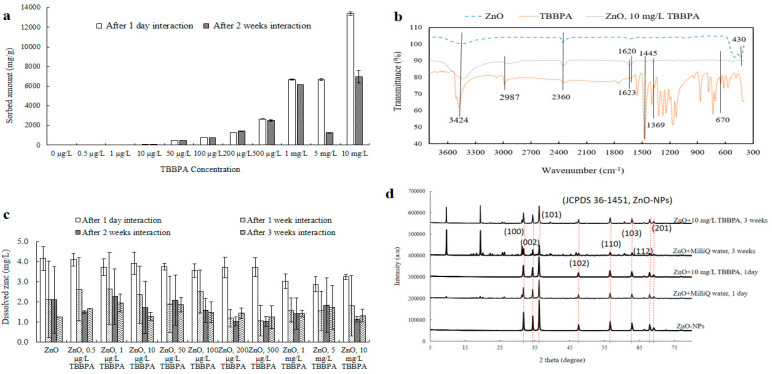
Adsorption (**a**), FTIR (**b**), dissolution (**c**), and XRD (**d**) analyses of ZnO-NPs after interaction with TBBPA after various time intervals.

**Figure 3:**
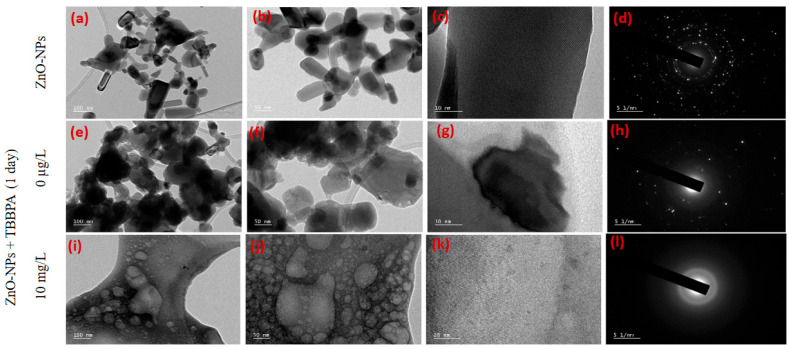
TEM analysis of the ZnO-NPs before and after one day of interaction with TBBPA. Bare ZnO-NPs (**a**–**d**), ZnO-NPs + 0 µg/L TBBPA after one day of interaction (**e**–**h**), and ZnO-NPs + 10 mg/L TBBPA after one day of interaction (**i**–**l**). From left to right, the scales of images are 100 nm, 50 nm, 20 nm and 5 1/nm.

Changes in the surface structure, particle dimensions, and ZnO-NPs’ surface charge were noted after they interacted with different concentrations of TBBPA. NPs’ electric potential and size were measured after one day, one, two, and three weeks of exposure (see Figure 1 and Figure S1). To verify interactions, TBBPA adsorption and FTIR analysis were conducted (refer to Figure 2a,b). The levels (concentrations, mg/L) of dissolved Zn were assessed both beforehand and following interactions with TBBPA (illustrated in Figure 2c). TEM analysis was carried out (after zero and one day of interaction) to examine the characteristics of NPs (depicted in Figure 3 and Figure S2).

Size and surface charge: Changes in both surface charge and particle size were noted after interacting with different concentrations of TBBPA. When varying amounts of TBBPA were added to zinc oxide NPs (one day), the peak of the particle size distribution curve (PSDC) for the ZnO-NPs remained within the range of 143 to 222 nm, while the overall particle size varied from 106 to 955 nm (Figure 1a and Figure S1a). Minor fluctuations in size at the peak of PSDC and across measured sizes after one day could result from the relatively short duration of contact between the nanoparticles and TBBPA. These results indicate that the interaction (ZnO-NPs + TBBPA) could depend on the duration of their coexistence. A notable elevation in size was perceived after a week of association (peak of PSDC rising to 166–222.5 nm). There was no observed upward trend in particle size from concentrations of 0.5 to 100 µg/L TBBPA. Nevertheless, there was a rise in the peak of size when the concentration of TBBPA rose from 200 µg/L to 10 mg/L. The variation in size distribution of nanoparticles was affected by TBBPA due to coating or adsorption of organic materials on the surface [24,25]. Throughout the interaction period, ZnO-NPs showed an increase in size, with notable growth observed (one day to three weeks). Enlargement could be due to the formation of large particles or subsequent sedimentation because of particles’ associations with each other. Initially, NPs were uniform in size following suspension preparation, but after one week of interaction, the particles became nonuniform (polydisperse). TBBPA may block the active sites of the nanoparticles, keeping them dispersed [50,51], which could be reflected by the ZnO-NPs’ sizes.

The analysis of TEM showed NPs’ dispersion and size reduction after 0 h and 1 day of interaction (refer to Figure 3). The particles observed between one and three weeks appeared in a polydisperse state, also indicated by their zeta potential (see Figure 1b). Adsorption analysis was conducted to measure the quantity of TBBPA that adhered to NPs, as detailed in the subsequent segment.

A decline in electrokinetic potential resulting from particle-to-particle interactions was noted from one day to three weeks of engagement. Nonetheless, the inclusion of different concentrations of TBBPA postponed the rapid reduction in electrokinetic potential over various periods. The changes in the electrical potential suggested that TBBPA covered the surface of the large structure of the ZnO-NPs and reversed their aggregation as the zeta potential increased. The values of zeta potential indicated that the ZnO-NPs showed increased stability in solution due to their distribution in the presence of TBBPA. This dispersion behavior could be linked to the extensive molecular structure of TBBPA, which likely coats the surface of the nanoparticles. This process is similar to the interaction between humic substances and NPs [25,26,27,28,52,53].

However, an increase in size was observed with a relatively small decrease in zeta potential from 500 µg/L to 10 mg/L of TBBPA after one week. With aging, the polydispersity of NPs increased (unequal attachment of the large molecular structure of TBBPA, affecting the overall size). TBBPA is a molecule with two hydroxyl groups and four bromine atoms attached to a central phenyl ring. Owing to the ionization characteristics of TBBPA in water [54], it can undergo ionization due to the presence of its hydroxyl groups. The presence of more hydroxyl groups due to higher TBBPA concentrations restrained the overall surface charge to a greater degree than their size. The diffraction pattern (Figure 3l) of ZnO-NPs + 10 mg/L TBBPA after one day was more amorphous (less crystalline) than that of ZnO-NP + 0 µg/L TBBPA (Figure 3h). However, the peak of PSDC of zinc oxide NPs + 0 µg/L TBBPA particles was smaller (such as 166 nm) than that of the ZnO-NP + 10 mg/L TBBPA particles (such as 222 nm), which could be due to the sorption of TBBPA molecules onto the surface of the nanoparticles, resulting in less crystallinity (amorphous) after one day of interaction. Notably, some particles were sedimented/attached to the walls of the tubes from the time of preparation of the mixtures (samples) to three weeks of interaction; hence, whatever was present in the suspension form was analyzed.

Adsorption, FTIR, dissolution, and XRD analyses: The adsorption of TBBPA indicated the presence of interactions between NPs and TBBPA after one day and two weeks of associations (Figure 2a). Quantity of TBBPA adsorbed onto NPs’ surface diminished after two weeks related to the amount observed after one day for higher concentrations (such as 500 µg/L, 1, 5, and 10 mg/L) of TBBPA. A range of interaction mechanisms may play a role in the adsorption of TBBPA onto the ZnO-NPs, including electrostatic interactions, hydrophobic interactions (such as π–π stacking, electron donor–acceptor interactions, and van der Waals forces), as well as hydrogen bonding [55].

When ZnO-NPs are exposed to water, hydrolysis commonly leads to generation of hydroxide layers ($\mathrm{Zn}{\left(\mathrm{OH}\right)}_{\left(\mathrm{aq}\right)}^{+}$) on the nanoparticle surface. This occurs as water molecules are adsorbed onto the particles both chemically and physically [56,57]. This could result in development of many positive charges on NPs’ surfaces, attracting deprotonated (TBBPA^−^) forms of TBBPA, which carry a negative charge to be sorbed on NPs’ surfaces. Initially (such as after one day of interaction), ZnO-NPs may have a high affinity for adsorbing TBBPA molecules because of the availability of active sites on their surface. However, the dispersion of NPs (polydispersed) due to adsorption of TBBPA (large molecular structure) led them to settle and decreased further adsorption with increasing time. Like HA, TBBPA may also form complexes with Zn ions released from the ZnO-NPs over time [58]. These complexes could alter the adsorption behavior of TBBPA and contribute to its desorption from the ZnO surface. This behavior resembles that of HA molecules, as the dispersion of ZnO-NPs might also be due to the complexation of zinc ions with anionic HA, leading to the creation of a larger complex structure [58] in which HA binds zinc ions.

FTIR analysis was performed on ZnO-NPs, TBBPA, and the ZnO + TBBPA mixture after one day of interaction to further investigate the interaction of TBBPA with the surface of the ZnO-NPs (Figure 2b). A peak at 430 cm^−1^ was observed, indicating Zn–O occurrence [28,59], which is typical of metal oxide spectra (400–600 cm^−1^) [60,61]. For pure TBBPA powder, a vibrational peak between 500 and 700 cm^−1^ was identified, corresponding to the stretching vibration of (C–Br) bonds in the organic pollutant. The 670 cm^−1^ peak was attributed to C–X stretching in organic halogen compounds, where X represents Br [62]. These peaks were also present in both TBBPA and ZnO-NPs after exposure to TBBPA [28,63]. Furthermore, peaks at 1145 and 1620 cm^−1^ were linked to C–O stretching and the skeletal vibration of aromatic C=C bonds within the TBBPA structure [63]. C–H bending and C=O stretching were detected at 1369 and 1623 cm^−1^, respectively [62,64]. Such peaks appeared in TBBPA and ZnO + TBBPA, confirming that bonding occurred between them. Additionally, peaks at 2987 cm^−1^ and 3424 cm^−1^ were associated with C–H/O–H stretching, C–H asymmetric stretching, and the water band [28,62]. FTIR data supported the existence of bonds in NPs after TBBPA interaction (one day), suggesting that TBBPA molecules accumulate on NPs’ surfaces. This observation aligns with the elemental analysis, which detected Br and C in the nanoparticles. The concentrations of dissolved zinc from NPs alone and those combined with different concentrations of TBBPA were assessed in this research (Figure 2c). Following a day of engagement, the levels of dissolved zinc increased, when TBBPA was present compared to when it was absent, and this pattern continued to be evident even after three weeks of interaction. It can be inferred that TBBPA facilitated the dispersion of NPs after an extended interface period (2–3 weeks).

Figure 2d shows the XRD study. NPs exhibited distinct peaks at 2θ values of 31.84°, 34.6°, and 36.5°, confirming NPs’ hexagonal wurtzite crystal structure with three specific orientations: (1 0 0), (0 0 2), and (1 0 1) [57]. These findings suggest that the crystal structure of NPs remained stable after exposure (one day). On the other hand, after three weeks of association (Figure 2d), peak strengths at (1 0 0), (0 0 2), and (1 0 1) decreased. Additionally, two crests appeared at 2θ angles of 9.68° and 19.40°, which might signify creation of different composites, zinc hydroxide dihydrate (Zn_5_(OH)_10_·2H_2_O) [65] and zinc phosphate [66], respectively. Peak intensities were lower for ZnO-NPs containing 10 mg/L of TBBPA compared to the sample that was in water, which could be because of TBBPA molecules covering ZnO-NPs’ surfaces (Figure 2d).

TEM: The initial ZnO nanoparticles (ZnO-NPs), obtained from Sigma-Aldrich, were agglomerated (size: 100 nm or less) with various shapes (see Figure 3a,b). The lattice pattern observed (Figure 3c) along with the glittering spots/rings (Figure 3d) confirmed the crystalline nature of the ZnO-NPs. The EDAX analysis conducted confirmed the occurrence of zinc and oxygen [28]. The diffraction pattern (Figure 3d) revealed the crystalline/lattice arrangement of the ZnO-NPs [28,67,68,69,70] which was comparatively dull/had fewer bright spots (Figure 3h) after their interaction than the original ZnO-NPs in powder form. The presence of both zinc and oxygen was also observed (Figure S2).

Following the interaction with TBBPA, the ZnO-NPs displayed highly random and dispersed structures (Figure 3i,j), in contrast with the original particle arrangement (Figure 3a,b). A high-resolution TEM image (Figure 3k) illustrated the dispersion pattern of the ZnO-NPs after exposure to the large and complex molecular structures of TBBPA (Figure 3c, and with 0 µg/L of TBBPA in Figure 3g). In contrast to the pure ZnO-NPs, cloudy spots (Figure 3l) indicated altered NPs’ morphology following their association with organic pollutant molecules (TBBPA). Elemental analysis indicated the existence of oxygen, zinc, bromine, and carbon atoms, as well as potassium and phosphorus, which came from the buffer (Figure S2).

### 3.2. Influence of Salts on the Interaction Between ZnO-NPs and TBBPA

Varying concentrations of cations and anions in ecosystem media can influence the physical and chemical characteristics of ZnO nanoparticles [19]. The stability of these systems is primarily determined by the charge present on the NPs’ surfaces. Environmental elements, such as pH, ionic strength, and the existence of organic materials in the solution, also have an impact on the surface charge. To assess their impact on NPs’ stability, both individually and in the existence of TBBPA, fixed concentrations (for instance, 5 mM) of NaCl and CaCl_2_ were utilized. The following section explains the outcomes.

**Figure 4:**
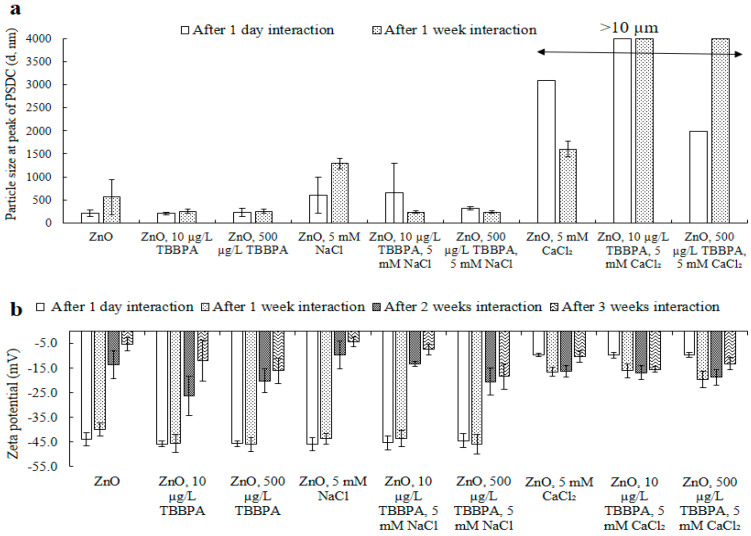
The size at the peak of PSDC for the ZnO-NPs (**a**) and zeta potential (**b**) after one day, one week, two weeks, and three weeks of interaction in the presence of electrolytes. The original particle sizes of the ZnO-NPs at the peak PSDC were 214 nm for one day and 560 nm for one week, and the zeta potentials were −43.9 mV for one day, −39.9 mV for one week, −13.7 mV for two weeks, and −5.3 mV for three weeks. Where, “-” represents minus sign in Figure 4b.

Hydrodynamic size: Changes in NPs’ sizes were recorded both in Milli-Q H_2_O and with TBBPA (at concentrations of 10 and 500 µg/L), as well as when combined with a consistent concentration (5 mM) of salts (NaCl and CaCl_2_), over periods of interaction (see Figure 4a and Figure S3). After one day, NPs’ size at the peak of PSDC enlarged with the occurrence of NaCl and CaCl_2_. This indicated that the salts had a predominant effect, likely due to the compression of the double layer resulting in a smaller hydrodynamic diameter and increased aggregation, even when TBBPA was also present. The introduction of 5 mM CaCl_2_ caused a significant increase in particle size, exceeding the upper limit of the zeta sizer’s measurement range (which goes up to 10 microns). Only the size of the measurable portion was reported (refer to Figure 4a and Figure S3).

Compared with that after one day, NPs’ size in buffered water was examined to increase after one week. However, no significant increase in size was examined in the presence of TBBPA. The presence of salts shifted NPs’ size after one week. Compared with NPs alone, NPs with the existence of NaCl (5 mM) revealed a similar and steady increase in particle size, as the size increased from 560 nm (NPs in Milli-Q H_2_O after one week) to 1290 nm (the size of the ZnO-NPs in the presence of 5 mM NaCl after one week). Various concentrations of CaCl_2_ had the same effect on the size of the ZnO-NPs. The measurable size has been reported (Figure 4a and Figure S3). These findings imply that NPs’ sizes grew as cations gathered on negatively charged NPs’ surfaces because of electrostatic forces. Hydrogen bonding and van der Waals forces further amplified this process, resulting in an overall increase in particle size [11,28,35].

The interaction of ZnO-NPs with low concentrations of TBBPA (e.g., 10 or 500 µg/L) was studied by introducing salts. The addition of these salts impacted the hydrodynamic properties of the ZnO-NPs, as more ions accumulated around the charged nanoparticles. Furthermore, when NaCl was present, the large TBBPA molecules coated and dispersed the nanoparticles after one week of exposure. NPs’ size alone varied greatly with time because of the aggregation/polydispersity effect. It was challenging to observe the size behavior of ZnO-NPs at environmentally relevant concentrations of co-contaminants over time, as evidenced by the measurement of TBBPA in solution after interaction with the nanoparticles.

Surface charge: The electrokinetic potential of zinc oxide NPs diminished from −43.9 mV to −39.9, −13.7, and −5.3 mV after intervals of 1 day, 1, 2, and 3 weeks, respectively (Figure 4b). The noticeable decline in NPs’ electric potential might be attributed to the agglomeration of multiple NPs due to hydrogen bonding, van der Waals forces, and hydrophobic interactions. A comparable reduction in the surface charge magnitude was noted in the presence of TBBPA (for concentrations of 10 and 500 µg/L). This zeta potential decrease was less pronounced than that observed with the ZnO-NPs, likely due to the dispersion effect resulting from TBBPA’s large molecular structure. Nevertheless, the aging effect also played a substantial role. Likewise, the introduction of salts modified the NPs’ electrokinetic potential (Figure 4b). It can be inferred that cation concentrations built up around the surfaces of nanoparticles that carried a negative charge, leading to a higher overall surface charge. A similar trend was noted regarding the dimensions of NPs, which grew larger in the existence of salts (Figure 4a and Figure S3).

**Figure 5:**
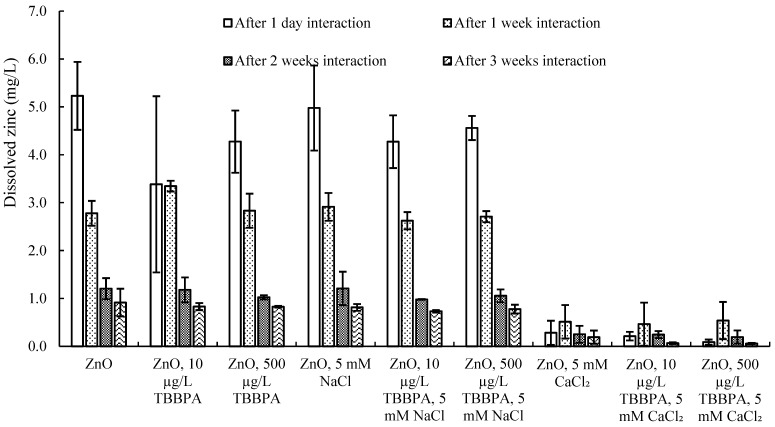
Dissolved zinc concentration (mg/L) after one day and after one, two, and three weeks of interaction.

Dissolution: The concentration of dissolved zinc (mg/L) was assessed using ICP–OES (Figure 5). Over several weeks of association, NPs’ size enhanced (aggregation followed by deposition), which led to a reduction in specific surface area and subsequently limited dissolution. Moreover, in the presence of CaCl_2_, a decrease in dissolution was noted compared to all other samples, potentially linked to increased agglomeration caused by the bridging effect of Ca^2+^. Additionally, nanoparticles tended to attach and settle within the low-density polyethylene tubes.

**Figure 6:**
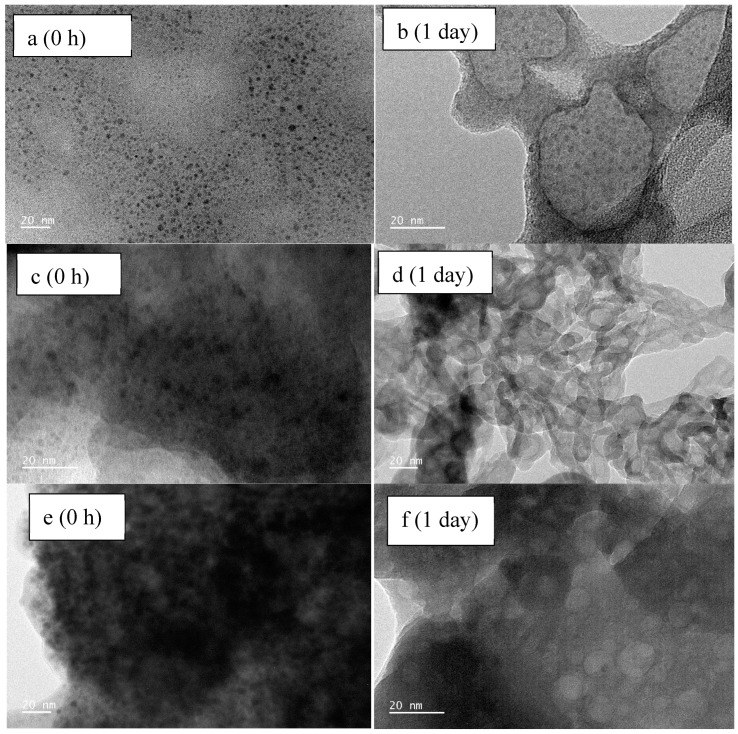
TEM images after 0 h and 1 day of interaction in solution (drops were taken on a TEM grid from the solution). ZnO, 10 mg/L TBBPA (**a,b**), ZnO, 10 mg/L TBBPA, 5 mM CaCl_2_ (**c**,**d**), ZnO, 10 mg/L TBBPA, and 10 mM CaCl_2_ (**e**,**f**).

TEM analysis: TEM analysis (Figure 6 and Figure S4) and elemental mapping (Figure S4) were conducted to examine the behavior of ZnO-NPs in the presence of TBBPA and varying concentrations of CaCl_2_ after 0 h and 1 day of interaction. A drop of the prepared solutions was directly placed on the TEM grid for observation. After one day of interaction, a dispersion effect, caused by the complex and aliphatic nature of TBBPA, was evident, with more nanoparticles being coated in the occurrence of 10 mg/L of TBBPA compared to 0 h (Figure 6a,b and Figure S4). This finding aligns with the electrokinetic potential and particle size data from the zeta analyzer (Figure 4a,b). SAED images revealed a reduction in crystallinity after one day compared to the initial time point. Elemental mapping also detected Zn, P, O, K, Br, and C (Figure S4). Aggregation with thick or shaded layers of CaCl_2_ was noted. After one day, both covered (CaCl_2_ coatings) and scattered (due to TBBPA) ZnO-NP were observed at both 5 and 10 mM CaCl_2_ concentrations. The diffuse diffraction patterns (Figure S4) could be attributed to the thick layers of TBBPA and salts on the nanoparticle surfaces. The bigger TBBPA molecules facilitated the NPs’ dispersion after one day.

### 3.3. Influence of HA on TBBPA and ZnO-NPs’ Interaction

**Figure 7:**
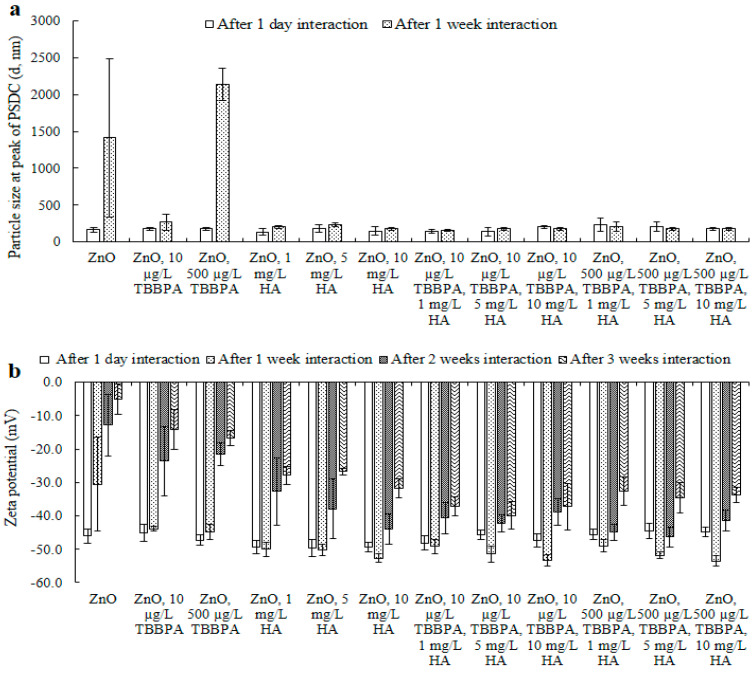
The size of ZnO-NPs at the peak of PSDC (**a**) and zeta potential (**b**) after one day and after one, two, and three weeks of interaction in the presence of various concentrations of TBBPA and HA at pH 7 and room temperature (i.e., 20 °C). The original particle size of ZnO-NPs at the peak of PSDC is 166 nm (one day) and 1413 nm (one week), and the zeta potential is −46.1 mV (one day), −30.5 mV (one week), −12.8 mV (two weeks), and −5.0 mV (three weeks). Where, “-” represents minus sign in Figure 7b.

Particle size: The size of ZnO-NPs in buffered water, exposed to varying concentrations of TBBPA (10 or 500 µg/L), different levels of HA (1, 5, or 10 mg/L), and combinations of TBBPA and HA, was assessed after 1 day and 1–3 weeks (Figure 7a and Figure S5). Size elevations were observed. This enlargement may be attributed to the formation of larger and/or sedimented particles, resultant from interactions between particles, as well as electrostatic and hydrophobic forces. Initially, the NPs were monodispersed. After one week, the nanoparticles displayed nonuniform characteristics (polydisperse). By the one to three weeks period, the particles were highly polydisperse, a pattern that was also revealed in their surface charge (Figure 7b). The distribution and dimensions of NPs in the occurrence of 1, 5, and 10 mg/L of humic acid diminished after one week of interaction, in contrast to the measurements recorded on day one (Figure 7a and Figure S5). After two and three weeks, samples became highly polydisperse, and their size exceeded the measurement range of the dynamic light scattering analyzer, as some samples exhibited scattering behavior. A similar trend was observed for ZnO-NPs exposed to different concentrations of TBBPA (10 and 500 µg/L), when combined with HA (Figure 7a and Figure S5). This dispersion is likely due to HA, with its large aliphatic carbon network, which may have capped the nanoparticle edges, promoting their dispersion. Coexistence of both TBBPA and HA influenced the NPs’ size in a manner distinct from the effect seen with either TBBPA or HA individually. The distribution and NPs’ size increased (resulting in agglomerated particles) when only ZnO-NPs or those in conjunction with TBBPA were present (an observable desorption pattern occurred over time (Figure 7a), which facilitated accumulation of NPs following several weeks of interaction). Conversely, when varying concentrations (1, 5, and 10 mg/L) of HA were present, NPs’ size decreased (leading to polydispersal). This dispersal behavior of NPs might be linked to the occurrence of HA, which could mitigate their aggregation tendencies [32,44].

Zeta potential: Electrokinetic potential results are shown (Figure 7b) and explained in this section. The strength of NPs’ electrokinetic potential, both independently and with TBBPA, showed a decline (one day to three weeks). Nevertheless, the decline in surface charge was less pronounced for the ZnO-NPs in the presence of TBBPA compared to those without it (Figure 7b). Additionally, the presence of HA led to a further reduction in zeta potential, in comparison to the samples without HA. A higher concentration of HA played a significant role in mitigating the reduction in electric potential, unlike lower concentrations of HA, such as 1 mg/L HA (Figure 7b) [31].

The previously mentioned data on electrical potential indicated that the clustering behavior of pure ZnO nanoparticles in water may stem from van der Waals forces, electrostatic interactions, and hydrophobic effects. NPs’ aging, both alone and alongside TBBPA, could modify the NPs’ electrical potential, resulting in increased sedimentation in water due to a reduction in stability in aqueous conditions. When comparing the effects of pure ZnO nanoparticles with and without TBBPA, the existence of humic acid changed the surface charge in an opposing manner. HA compounds covered the NPs’ surfaces and effective sites due to their higher aliphatic carbon content compared to TBBPA molecules, which led to a reduced likelihood of TBBPA adhering to the surfaces of the nanoparticles. This also promoted better dispersion of the nanoparticles.

**Figure 8:**
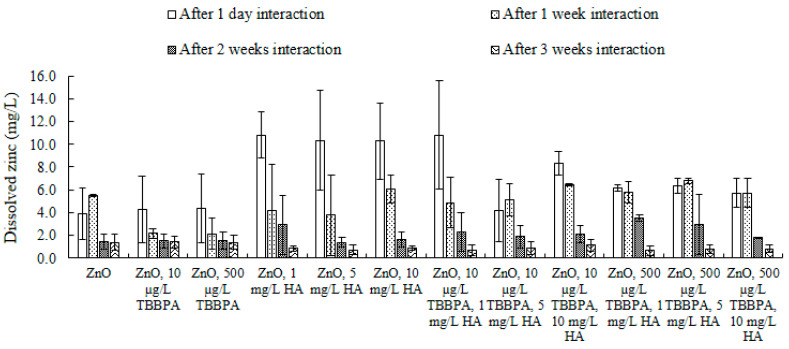
Dissolved zinc concentration (mg/L) after one day and after one, two, and three weeks of interaction in the presence of various concentrations of TBBPA and HA at pH 7 and room temperature (i.e., 20 °C).

Dissolution: ZnO-NPs’ dissolution, whether by themselves or alongside different concentrations of TBBPA, HA, and their combination, was examined in Milli-Q water at a pH of 7 (Figure 8). The availability of zinc, either in its dissolved state or as an ionic form, poses potential toxicity to microorganisms, including microflora [58,71]. The existence of additional compounds in water can affect the dissolution of ZnO-NPs [72]. After one day, concentration of dissolved zinc was higher when various concentrations of HA were present compared to when HA was absent, and this pattern continued even after two weeks of interaction. It can be postulated that HA facilitated the dispersion of the nanoparticles after extended interaction periods (like two to three weeks), and the increased dispersion might result from van der Waals forces, electrostatic forces, and hydrophobic interactions. Additionally, this might be attributed to the complexation of zinc ions with the anionic HA, leading to the formation of a larger complex structure. These findings align with those posited in [58] that HA binds zinc ions.

### 3.4. Influence of BSA on the Behavior of ZnO-NPs

**Figure 9:**
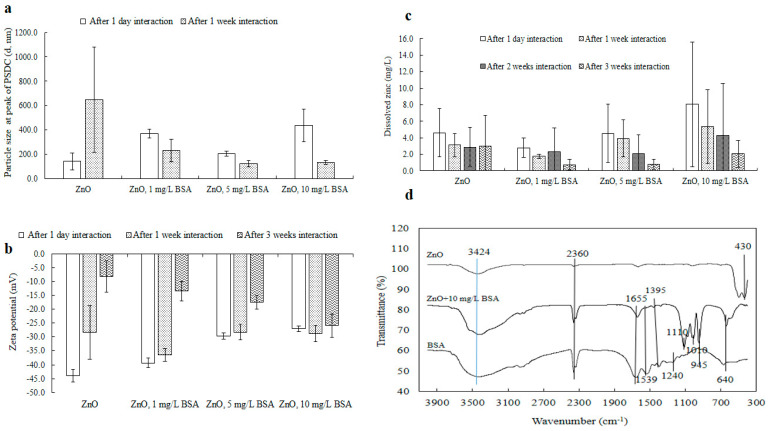
ZnO-NPs’ sizes at the peak of PSDC (**a**), zeta potential (**b**) (after one day, one week, and three weeks), dissolution (**c**) (after one day, one week, two weeks, and three weeks), and FTIR (**d**) (after one day) of interactions with BSA. The original particle sizes of the ZnO-NPs at the peak PSDC were 140.7 nm after one day and 648.5 nm after one week, and the zeta potentials were −44.0 mV after one day, −28.3 mV after one week, and −8.0 mV after three weeks. Where, “-” represents minus sign in Figure 9b.

The effect of bovine serum albumin (BSA), a natural protein found in ecological water, on the stability of ZnO-NPs was investigated (Figure 9a,b and Figure S6). BSA plays various physiological roles, including transporting, binding, and distributing fatty acids and steroids [73]. In this study, BSA was selected as a model protein due to its water-soluble properties.

Hydrodynamic size: Initially, after one day, NPs’ sizes increased in the presence of BSA (Figure 9a). The BSA molecule, with a large molecular mass of 66,400 Da and consisting of approximately 583 amino acids linked in a single cross-linked chain with 17 cysteine residues [74], contributed to this increase. However, after one week of incubation, the presence of BSA reduced the size of the ZnO-NPs. The resulting dispersion effect was similar to that observed when other large molecular materials, like humic acid [28], interacted with the nanoparticles. As the concentration of BSA increased, NPs’ sizes, measured at the peak of the PSDC, gradually decreased (Figure 9a and Figure S6b). This behavior is consistent with the typical interactions between BSA and metal ions, which can lead to a decrease in the protein’s configuration due to the disruption of disulfide bonds. This results in a partial loss of the α-helix structure, unfolding of the protein, or changes in the polarity of the surrounding environment, which may affect the exposure of tryptophan residues due to molecular interactions. These reactions include excited-state processes, molecular adjustments, energy transfer, complex formation, or collision quenching [74].

**Figure 10:**
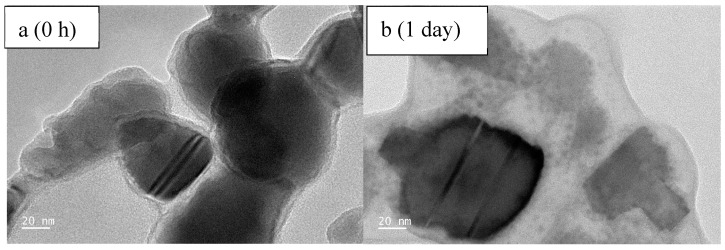
TEM images of ZnO + 10 mg/L BSA after 0 h (**a**) and 1 day (**b**) of interaction in solution (drop taken on a TEM grid from the solution).

Zeta potential: Figure 9b shows the surface charge values on the surface of the ZnO-NPs before and after interactions with BSA at various time intervals. Compared with that after one day, the overall surface charge (magnitude) of the ZnO-NPs diminished after several weeks of interaction. Similarly, NPs’ electrical potential with varying concentrations of BSA also decreased in magnitude after one day (Figure 9b), aligning with the findings from the particle size analysis (e.g., the size increased after one day; Figure 9a). This suggests that BSA, as a frothy substance, quickly coated the nanoparticles, causing the formation of large clusters, as confirmed by TEM analysis (only for 0 h and 1 day of interaction; Figure 10 and Figure S7). However, after several weeks of interaction, the surface charge did not decrease compared with that of the ZnO-NPs, implying that the dispersion revealed the protein patterns of the BSA molecules. These findings were further supported by the size (Figure 9a) and TEM (Figure 10 and Figure S7) analyses. The sizable and intricate molecular structure of the BSA protein molecules tended to envelop the ZnO-NPs, causing their dispersion, which in turn influenced their zeta potential.

Dissolution: The NPs’ dissolution was evaluated both on its own and in conjunction with different concentrations of BSA in Milli-Q water at a pH of 7 (Figure 9c). After a day, the dissolved zinc concentration was higher when BSA was present at various levels (such as 5 and 10 mg/L) compared to when it was absent, and this pattern continued even after two weeks. It can be inferred that BSA aided in dispersing the nanoparticles following a prolonged interaction period (of 2–3 weeks), and the enhancement in this dispersion may be attributed to electrostatic forces, van der Waals interactions, and hydrophobic forces.

FTIR: FTIR spectra of ZnO-NPs with BSA were examined after one day (Figure 9d). The peak at 430 cm^−1^ in the spectrum of the pure ZnO-NPs confirmed the existence of Zn–O [28,59]. The peak at 430 cm^−^^1^ represented the existence of metal oxides (such as ZnO). This peak was visible for pure ZnO-NPs (Figure 9d). However, after interactions with BSA, the intensity of the peak was not detectable by infrared spectroscopy. It could be assumed that BSA molecules (10 mg/L) adsorbed on NPs’ surface and generated coated layers that may have affected the detection of the ZnO-NP peak at 430 cm^−^^1^. As a result, the BSA layer might absorb or scatter the incident light in a way that reduces/weakens the intensity of the ZnO peak at 430 cm^−1^. The peak at 640 cm^−1^ in both samples was attributed to the secondary amide (N–H) [62] present in the BSA molecules. Notable bands in BSA included amide III at 1240 cm^−1^, which was not observed at the same peak location in the ZnO + BSA sample, amide II at 1539 cm^−1^, and amide I at 1655 cm^−1^ [75]. This could be due to the presence of interacting BSA molecules on NPs’ surface. The peaks observed at 945 and 1010 cm^−1^ could be attributed to the stretching of metal (zinc) and nitrogen bonds present in ZnO + 10 mg/L BSA after their interaction [62]. The peaks at 1110 cm^−1^ and 1395 cm^−1^ likely corresponded to C–O stretching and C–H stretching found in the organic BSA protein [62]. The peaks at 2360 cm^−1^ and 3424 cm^−1^ were associated with C–H/O–H stretching, C–H asymmetric stretching, and the water band, respectively [28,62]. These observations suggest that BSA interacted with NPs’ surfaces through π–π stacking as well as other molecular forces, including electrostatic and van der Waals interactions. The interactions further involved hydrophobic π–π stacking and hydrogen bonding between the active sites, such as oxygen-functionalized groups in water and oxygen/nitrogen groups within the protein molecules [76].

TEM: To assess the aggregation and dispersion behavior of ZnO-NPs in the presence of BSA at different time points, a TEM analysis was conducted by placing a drop of the solution directly onto the TEM grids (Figure 10 and Figure S7). Initially, at 0 h, the nanoparticles remained undispersed, likely due to the binding forces exerted by the proteins, aided by hydrogen bonding and electrostatic and hydrophobic interactions. However, after one day of interaction with BSA molecules, the ZnO-NPs were observed to disperse (Figure 10 and Figure S7), which could be attributed to the extensive coverage of nanoparticles by BSA molecules. Diffraction images (Figure S7) revealed bright spots at 0 h, indicating the crystalline nature of the ZnO-NPs, while after 1 day, the patterns appeared diffuse and cloudy, suggesting a reduction in NPs’ purity and crystallinity due to the interaction with BSA. Elemental mapping (Figure S7) at both time points confirmed the presence of nitrogen, oxygen, zinc, and carbon in the ZnO + 10 mg/L BSA samples. The presence of potassium (K) and phosphorus (P) was traced back to the buffer solution, which was used to maintain the pH at 7.

## 4. Conclusions

This research illustrated the surface and structural characteristics of ZnO-NPs under different environmental conditions, both before and after their interaction with co-occurring electrolytes, an organic pollutant (TBBPA), HA, and BSA over different periods, including one day, one week, two weeks, and three weeks of interaction. After engaging with environmental agents, ZnO-NPs were not found in their original forms due to alterations in particle size and shape. The inclusion of electrolytes enhanced the aggregation of charged ZnO-NPs by reducing the level of surface charge. The interaction mechanisms could be attributed to electrostatic forces, van der Waals forces, and particle–particle interactions, such as cation bridging. The large molecular structures of HA, BSA, and TBBPA contributed to a decrease in the particle size of the ZnO-NPs due to a dispersion effect. Changes in the shape, size, and surface charge of the ZnO-NPs were noted following their interaction with the co-contaminants, affecting the dynamics and behavior of the ZnO-NPs in aquatic environments.

## Data Availability

The original contributions presented in this study are included in the article/Supplementary Materials. Further inquiries can be directed to the corresponding author(s).

## Supplementary Materials

The following supporting information can be downloaded at: https://www.mdpi.com/article/10.3390/toxics13030148/s1. Figure S1: Particle size distribution of ZnO-NPs after 1 day (a) and 1 week (b) of interaction with TBBPA; ZnO-NPs 0.1 g/L, pH 7. Figure S2: Compositional analysis of ZnO-NPs with TBBPA after 1 day of interaction. Figure S3: Particle size distributions of the ZnO-NPs after 1 day (a) and 1 week (b) of interaction in the presence of electrolytes at pH 7 and room temperature (i.e., 20 °C). Figure S4: TEM images (elemental mapping) of various contaminants after 0 h and 1 day of interaction in solution (drop taken on a TEM grid from the solution). Figure S5: Particle size distributions of the ZnO-NPs after 1 day (a) and 1 week (b) of interaction in the presence of various concentrations of TBBPA and HA at pH 7 and room temperature (i.e., 20 °C). Figure S6: Particle size distribution of the ZnO-NPs after 1 day (a) and 1 week (b) of interaction in the presence of various concentrations of BSA at pH 7 and room temperature (i.e., 20 °C). Figure S7: TEM images (elemental mapping) of ZnO + 10 mg/L BSA after 0 h and 1 day of incubation in solution (drops taken on a TEM grid from the solution).
