# Supplementary material for: Influence of Tetrabromobisphenol-A on the Fate and Behavior of Zinc Oxide Nanoparticles Affected by Salts, Humic Acid, and Bovine Serum Albumin in Water Systems
Source: Toxics. 2025 Feb 21;13(3):148. doi: 10.3390/toxics13030148 (PMC11945663; doi:10.3390/toxics13030148)
Supplement: Supplementary file 1 [file toxics-13-00148-s001.zip › toxics-3453378-supplementary.pdf]

Influence of tetrabromobisphenol-A on the fate and behavior of zinc oxide nanoparticles affected by salts, humic acid and bovine serum albumin in water systems

Anwar Ul Haq Khan[1,2], Yanju Liu[1,2*], Ravi Naidu[1,2], Cheng Fang[1,2], Ho Kyong Shon[3]

[1]Global Centre for Environmental Remediation (GCER), College of Engineering Science and Environment, The University of Newcastle, Callaghan, NSW 2308, Australia

[2]crc for Contamination Assessment and Remediation of the Environment (crcCARE), ATC Building, The University of Newcastle, Callaghan, NSW 2308, Australia

[3]School of Civil and Environmental Engineering, University of Technology Sydney (UTS), City Campus, Broadway, NSW 2007, Australia

- Corresponding author: Dr. Yanju Liu, yanju.liu@newcastle.edu.au

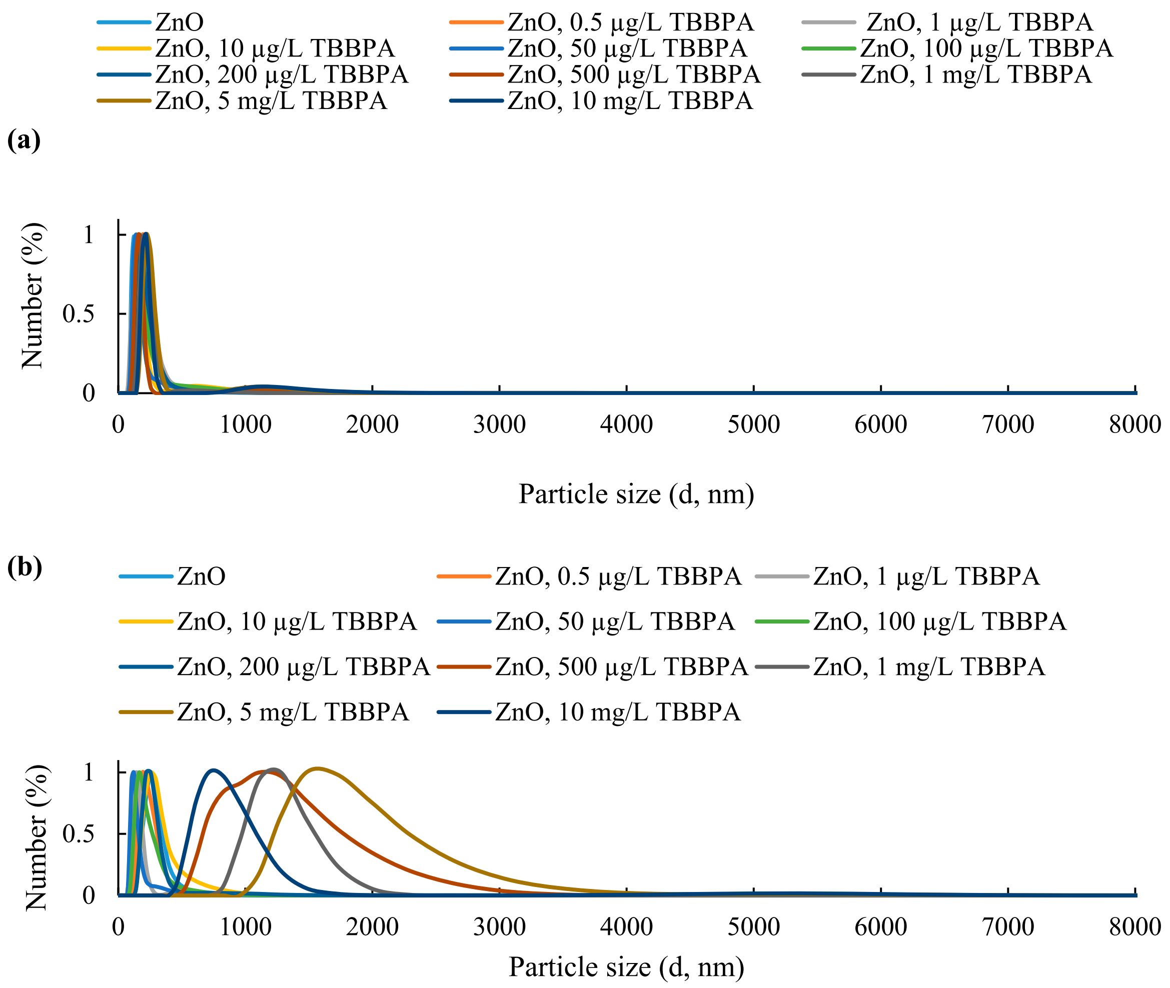

Figure S1 Particle size distribution of ZnO-NPs after 1 day (a) and 1 week (b) of interaction with TBBPA; ZnO-NPs 0.1 g/L, pH 7.

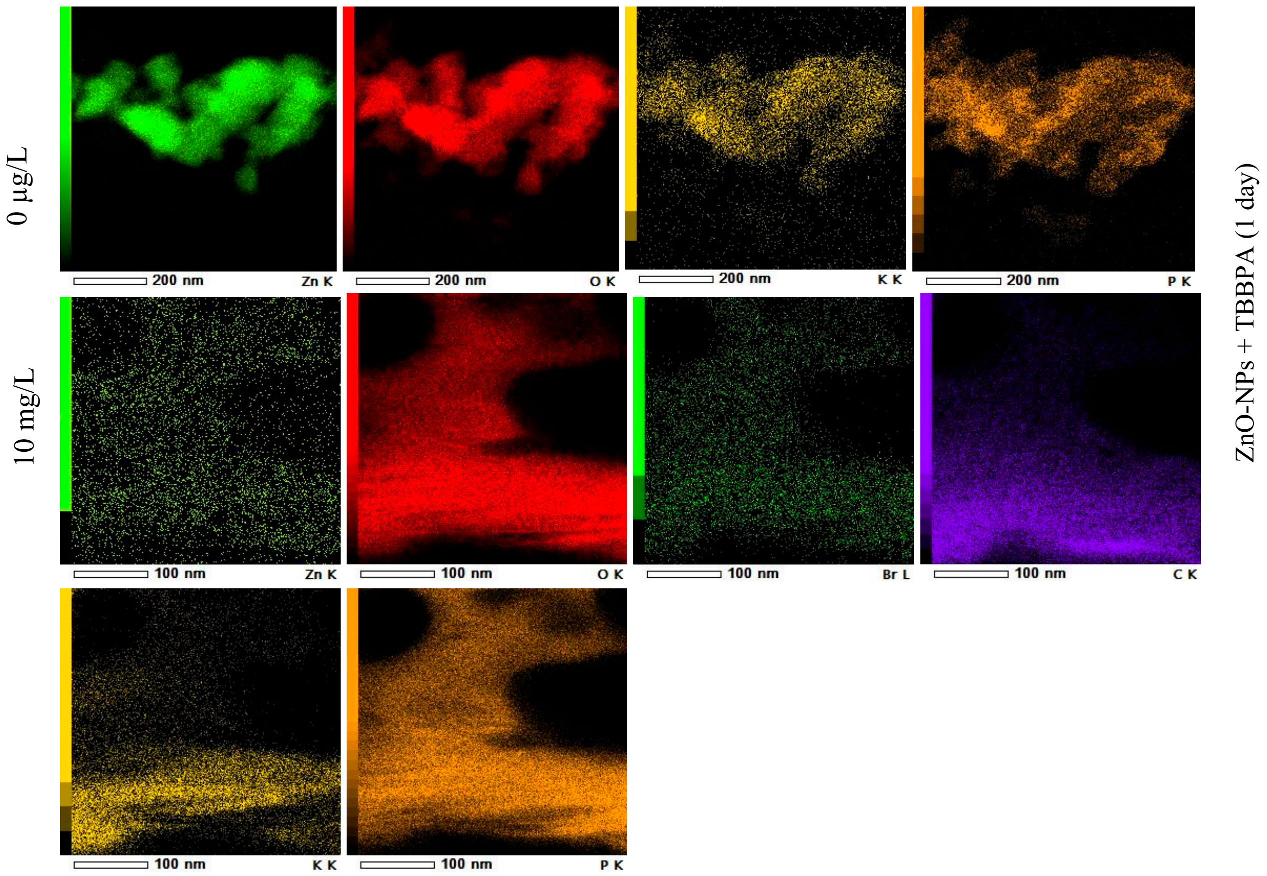

Figure S2 Compositional analysis of ZnO-NPs with TBBPA after 1 day of interaction.

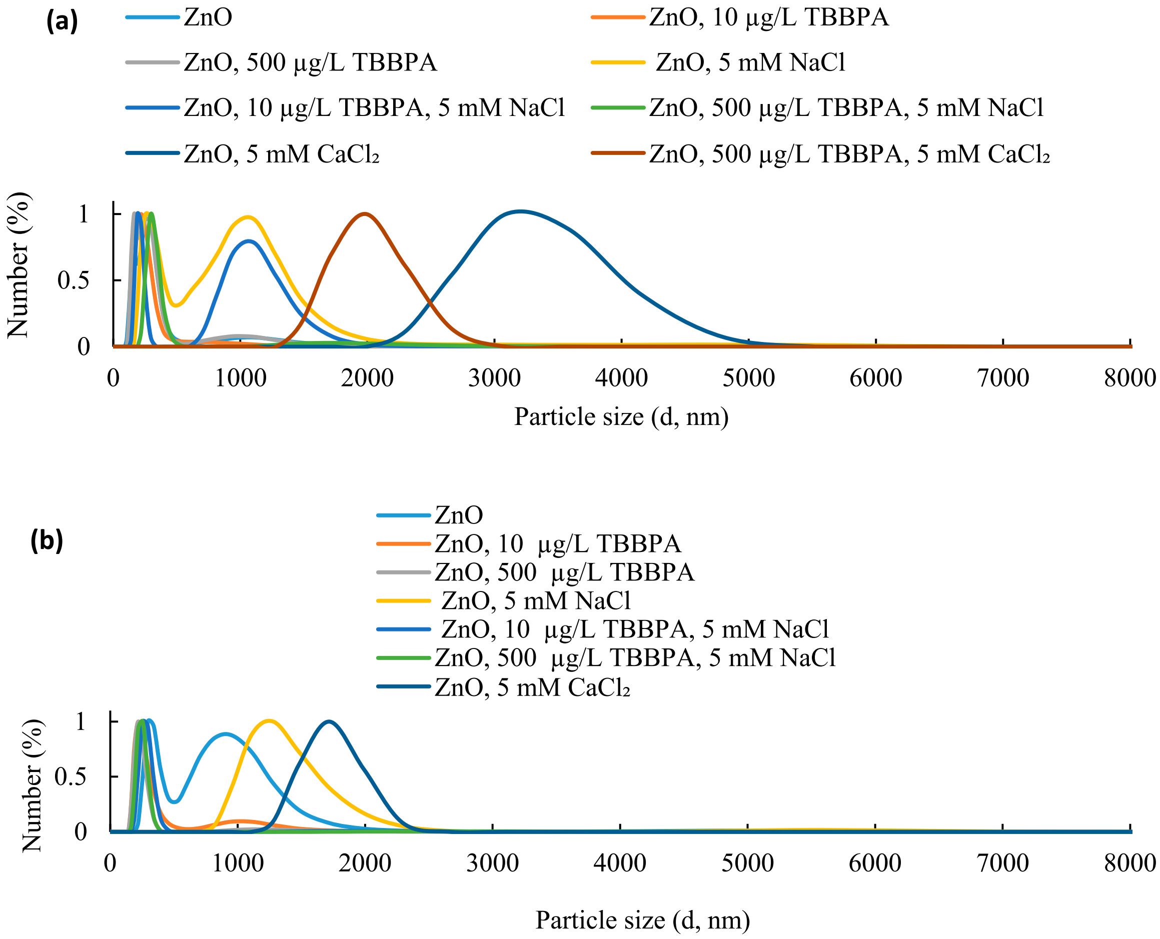

Figure S3 Particle size distributions of the ZnO-NPs after 1 day (a) and 1 week (b) of interaction in the presence of electrolytes at pH 7 and room temperature (i.e., 20 °C).

Figure S4 TEM images (elemental mapping) of various contaminants after 0 h and 1 day of interaction in solution (drop taken on a TEM grid from the solution).

<table>
<tr><th>Contaminants</th><th>0 h interaction</th><th>1 day interaction</th></tr>
<tr><td>ZnO, 10 mg/L TBBPA</td><td>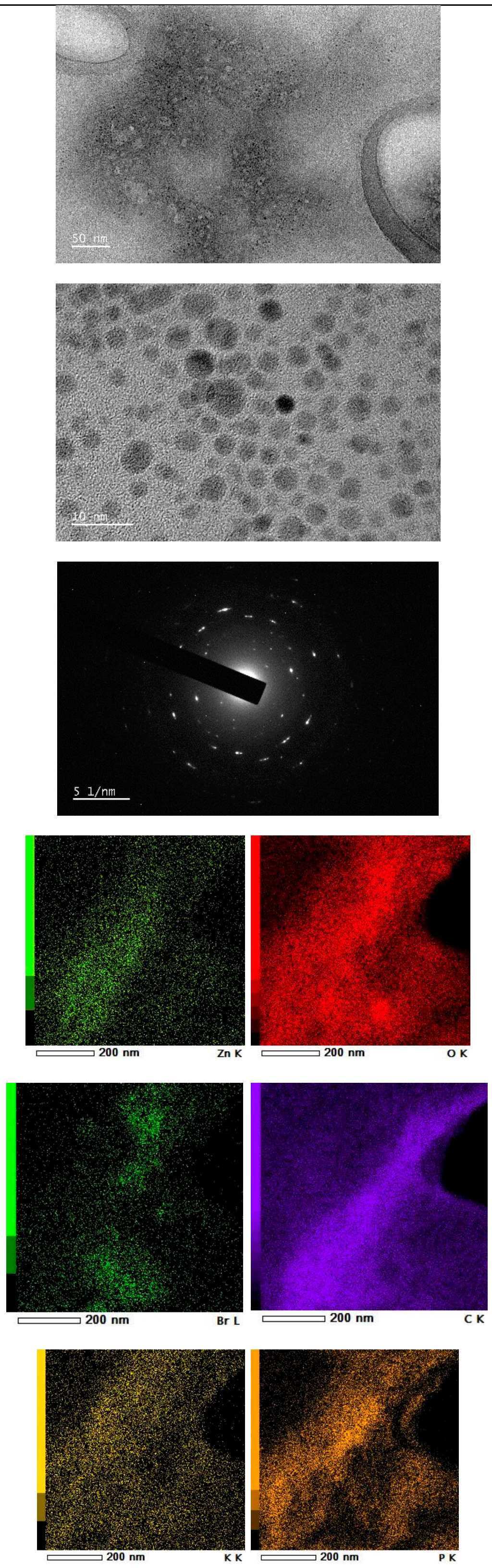
</td><td>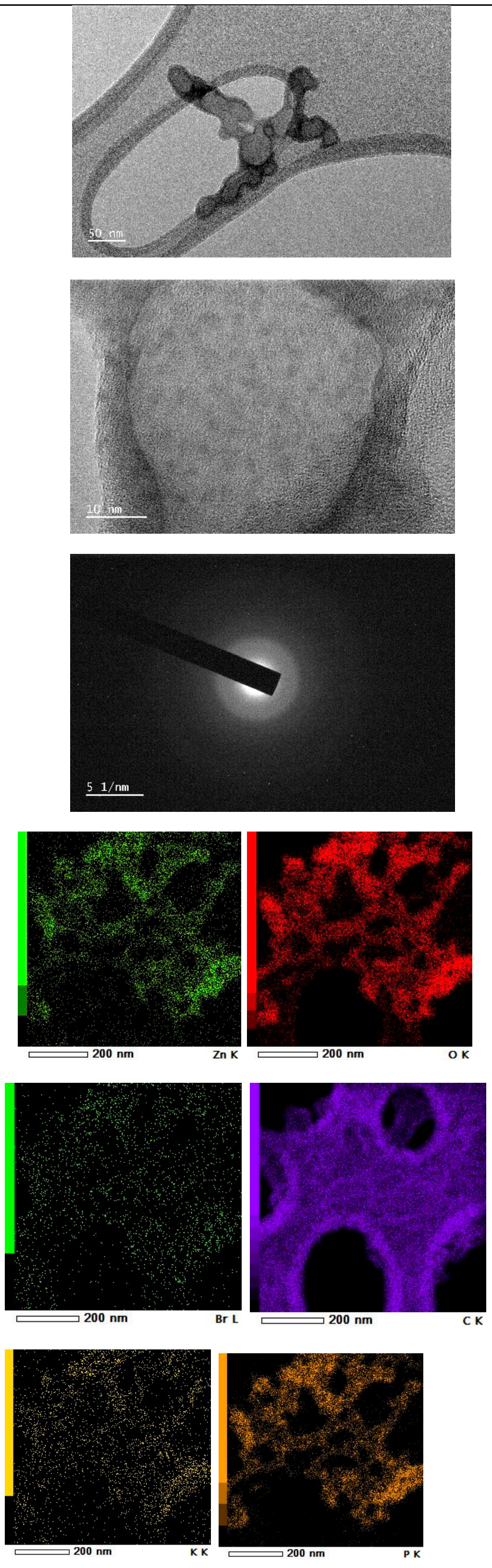
</td></tr>
</table>

ZnO, 10 mg/L TBBPA, 5 mM $CaCl_2$	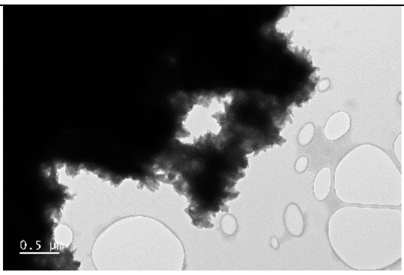<br>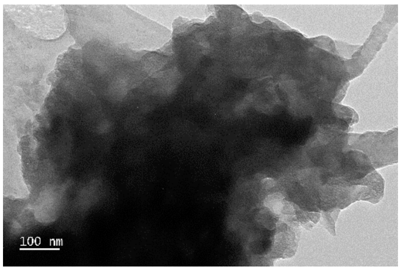<br>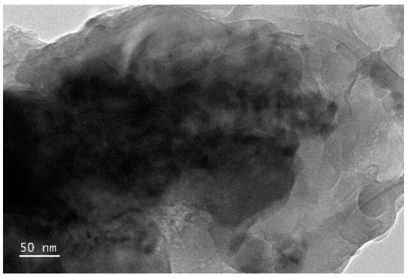<br>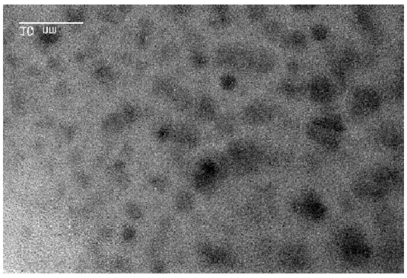<br>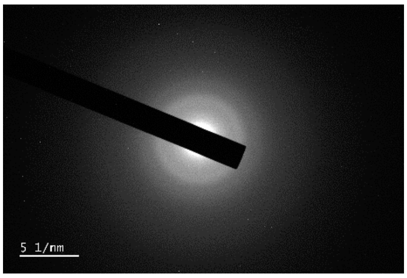<br>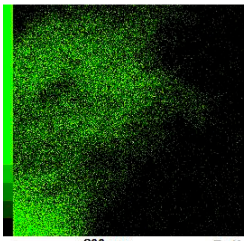<br>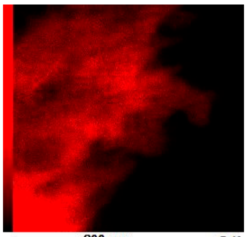	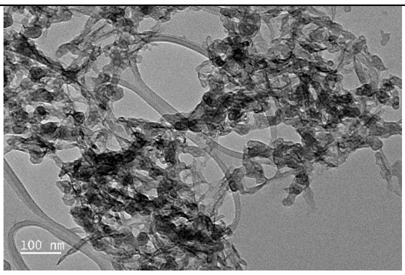<br>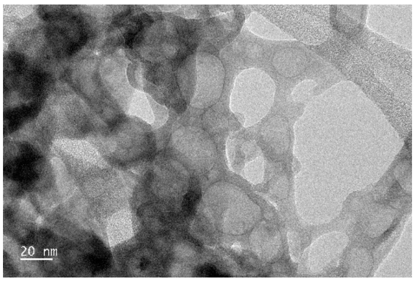<br>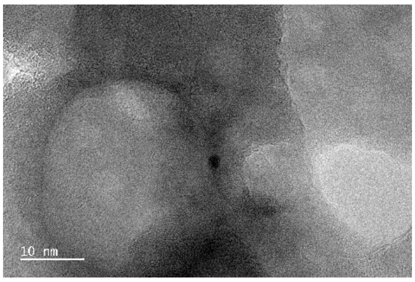<br>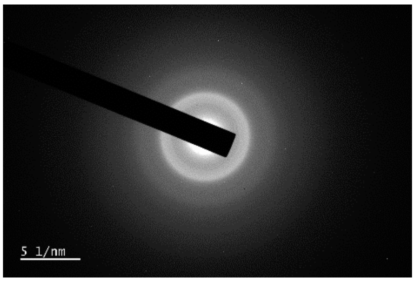<br>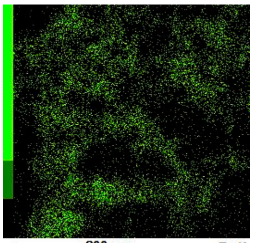<br>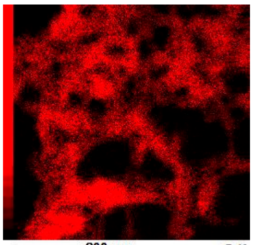<br>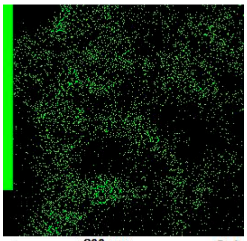<br>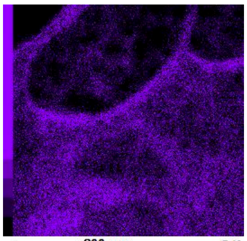

	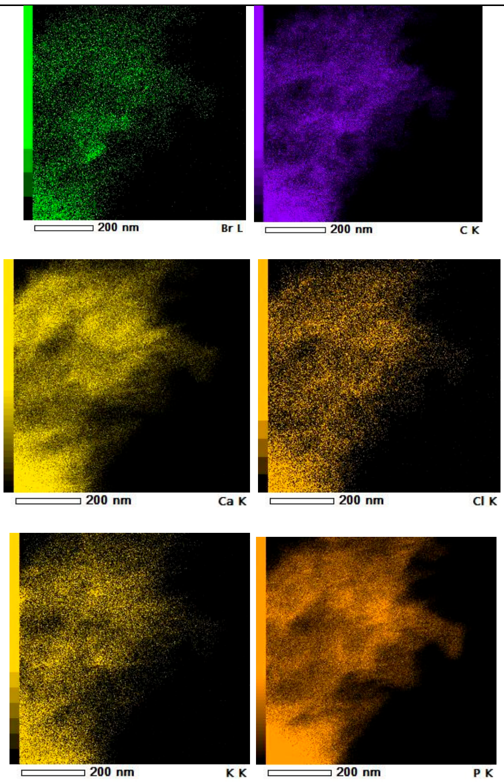	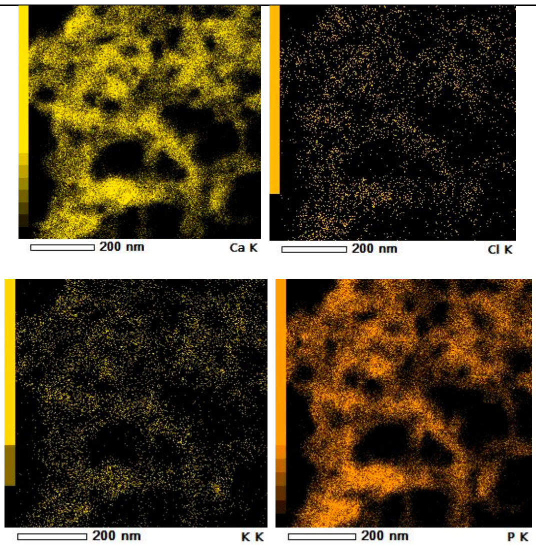
ZnO, 10 mg/L TBBPA, 10 mM $CaCl_2$	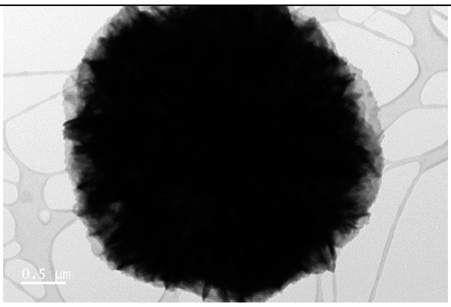<br>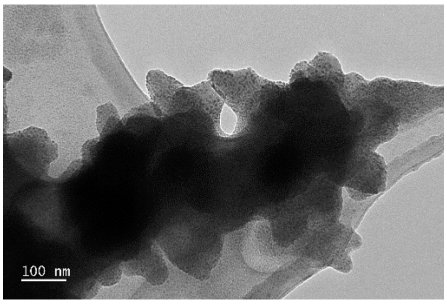<br>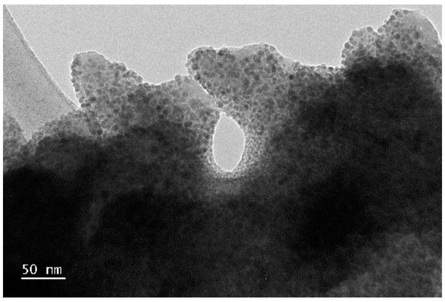	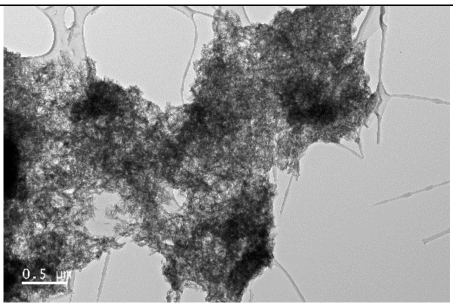<br>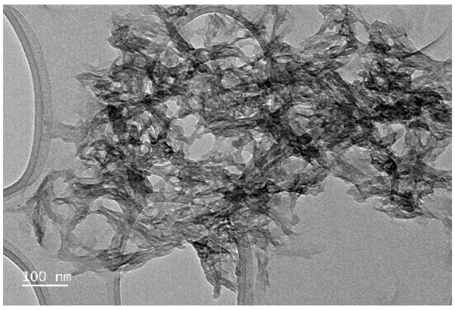<br>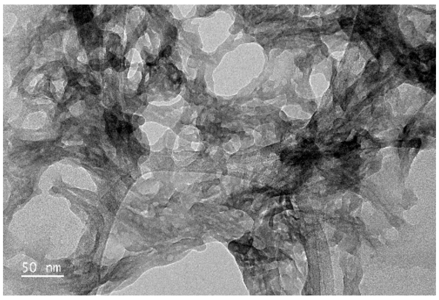

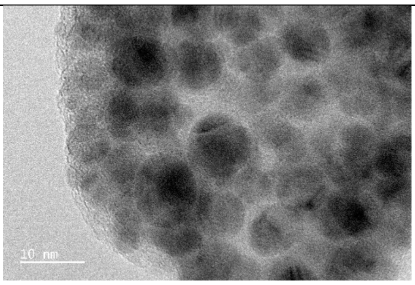
10 nm

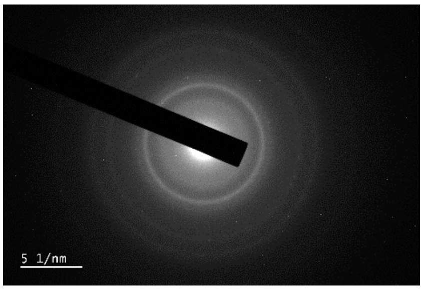
5 1/nm

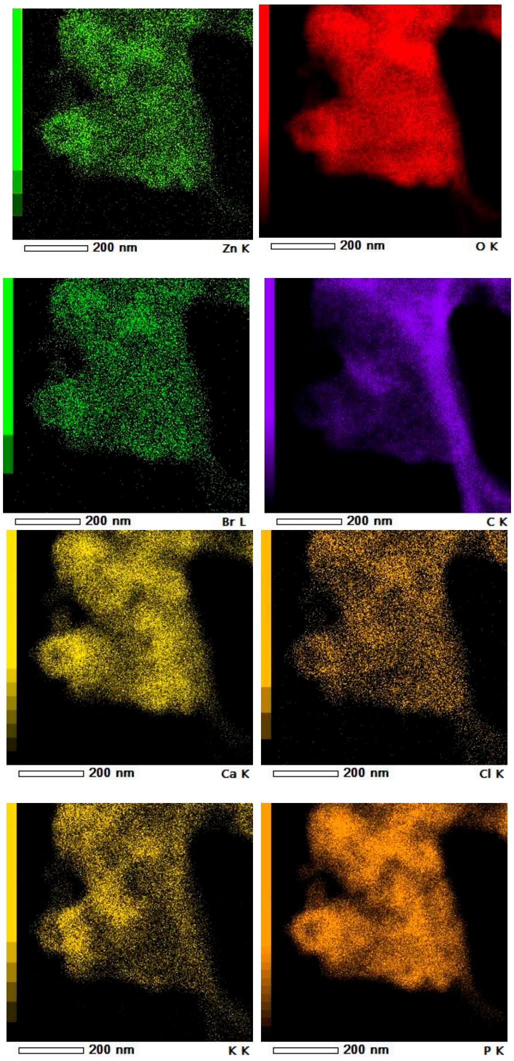
200 nm
Zn K
200 nm
O K
200 nm
Br L
200 nm
C K
200 nm
Ca K
200 nm
Cl K
200 nm
K K
200 nm
P K

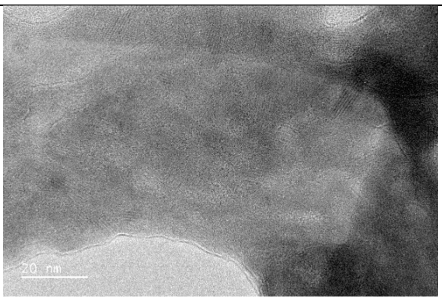
20 nm

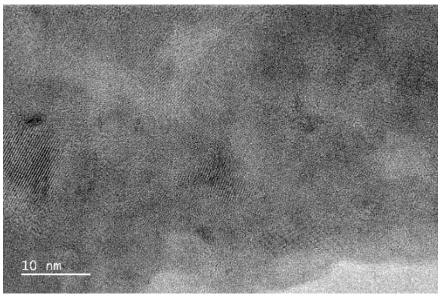
10 nm

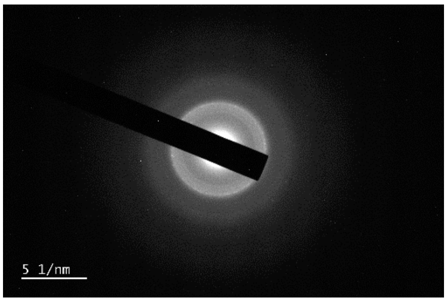
5 1/nm

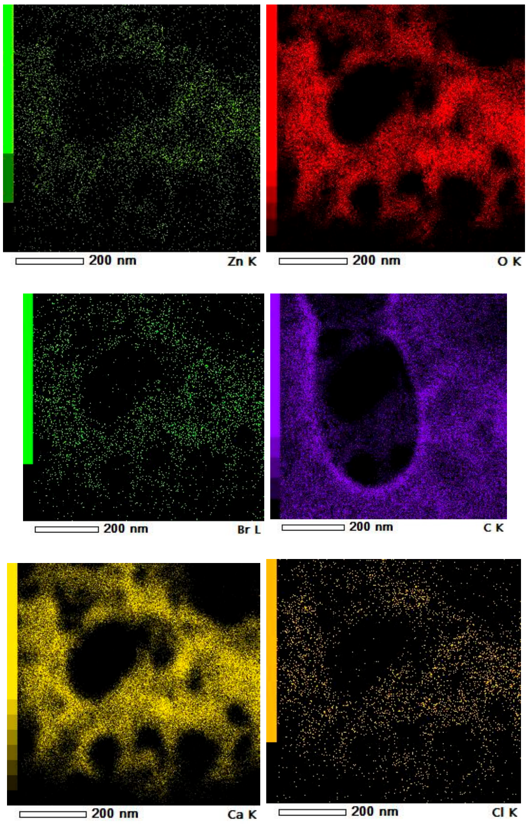
200 nm
Zn K
200 nm
O K
200 nm
Br L
200 nm
C K
200 nm
Ca K
200 nm
Cl K

		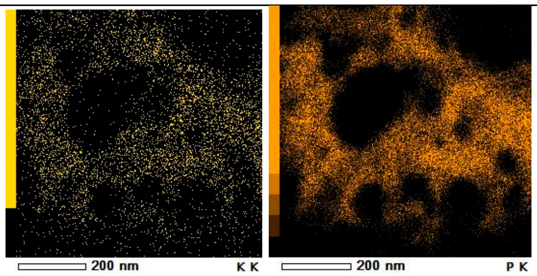 200 nm<br>K K<br>200 nm<br>P K

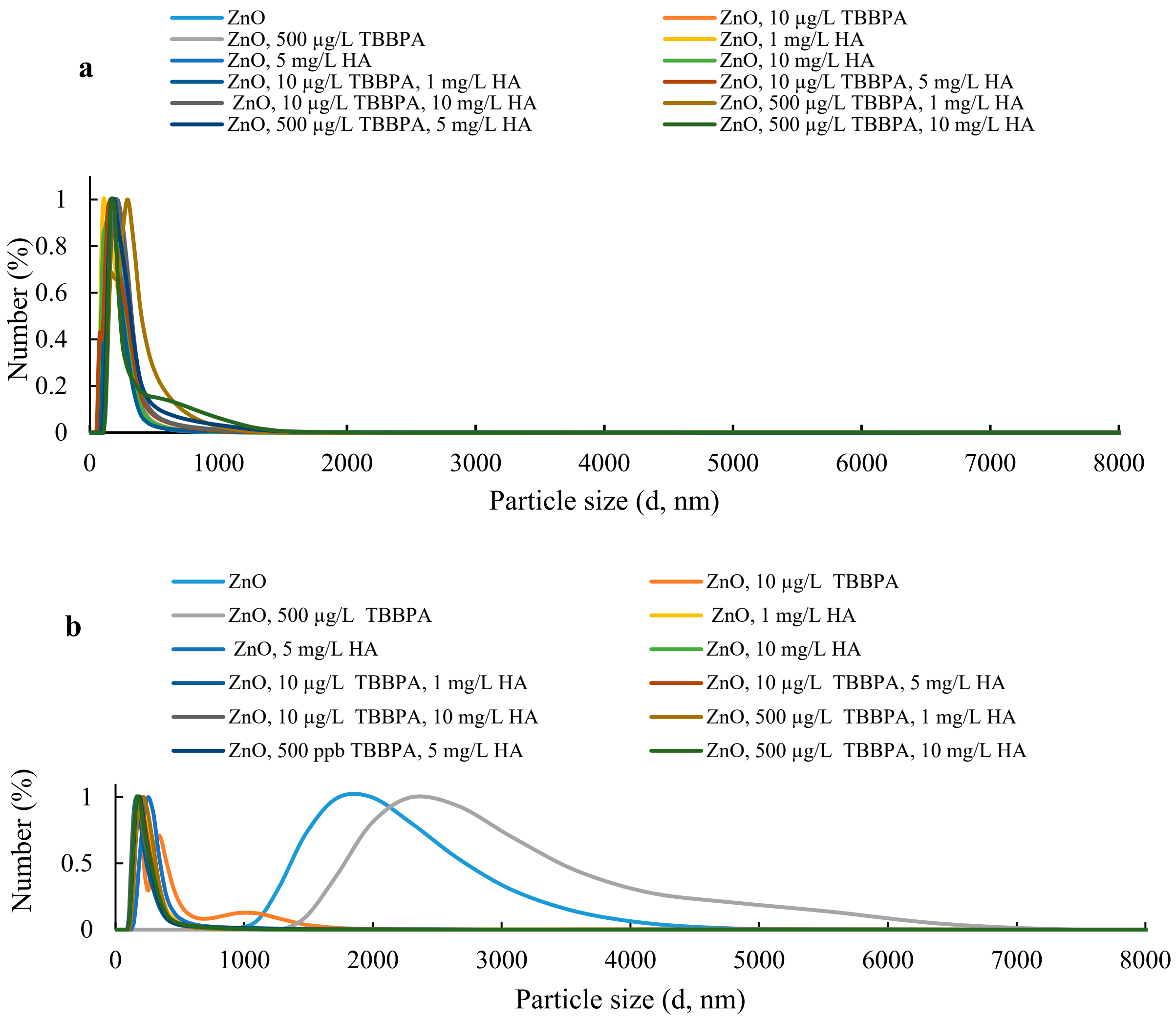

Figure S5 Particle size distributions of the ZnO-NPs after 1 day (a) and 1 week (b) of interaction in the presence of various concentrations of TBBPA and HA at pH 7 and room temperature (i.e., 20 °C).

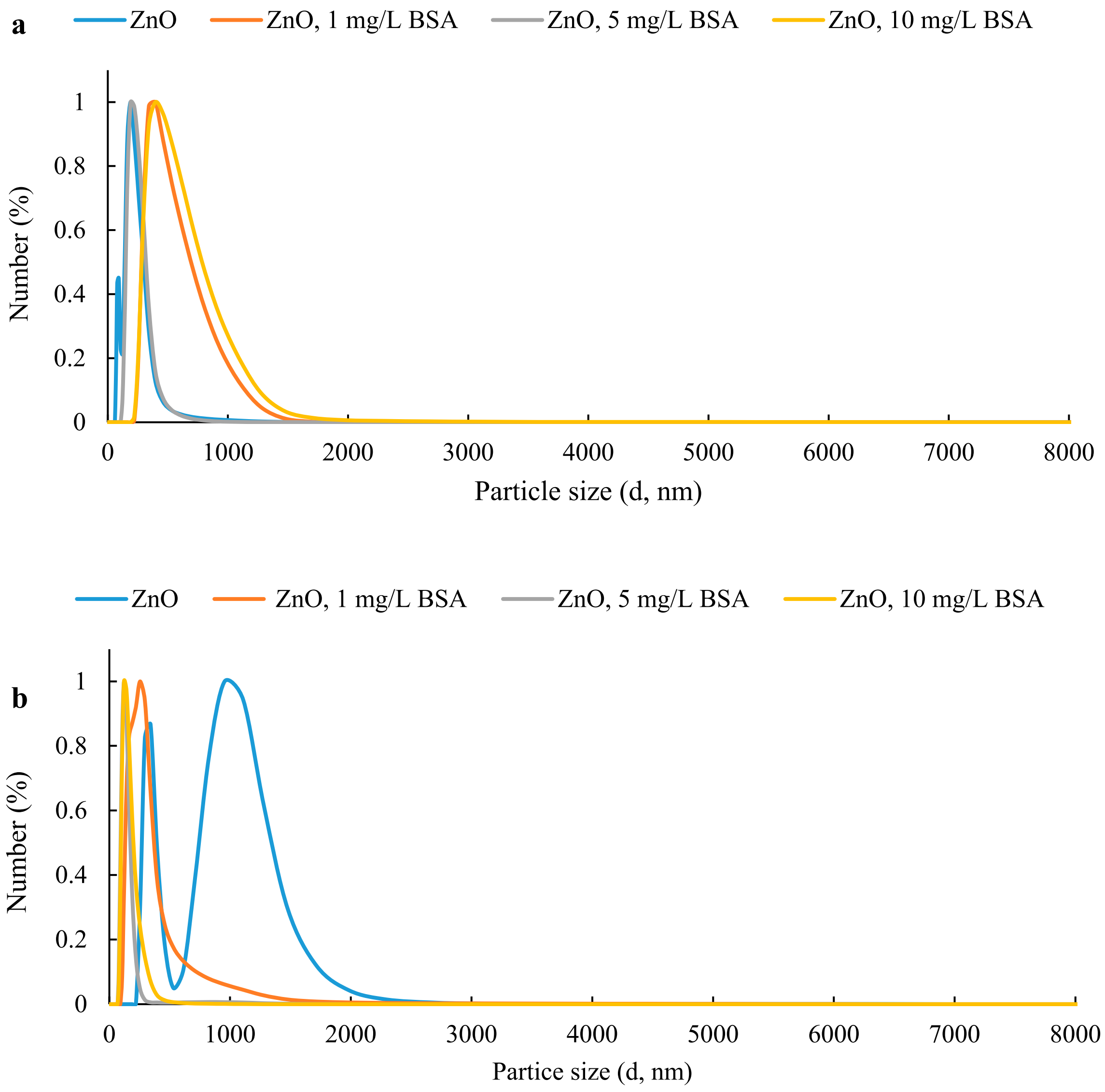

Figure S6 Particle size distribution of the ZnO-NPs after 1 day (a) and 1 week (b) of interaction in the presence of various concentrations of BSA at pH 7 and room temperature (i.e., 20 °C).

Figure S7 TEM images (elemental mapping) of ZnO+10 mg/L BSA after 0 h and 1 day of incubation in solution (drops taken on a TEM grid from the solution).

Contaminants	0 h interaction	1 day interaction
ZnO + 10 mg/L BSA	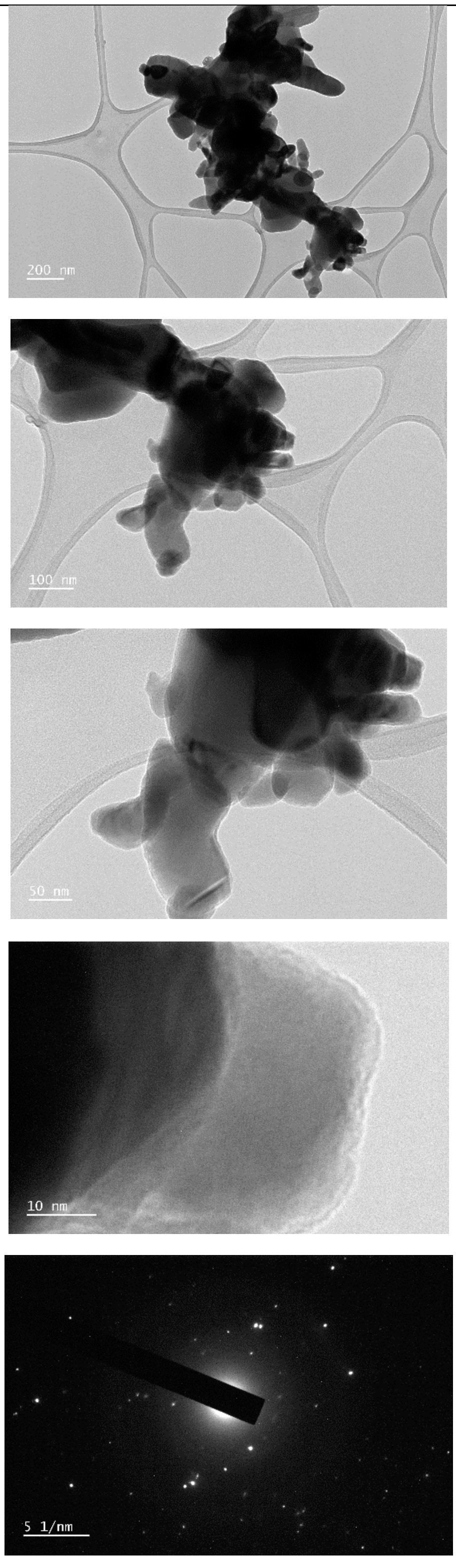	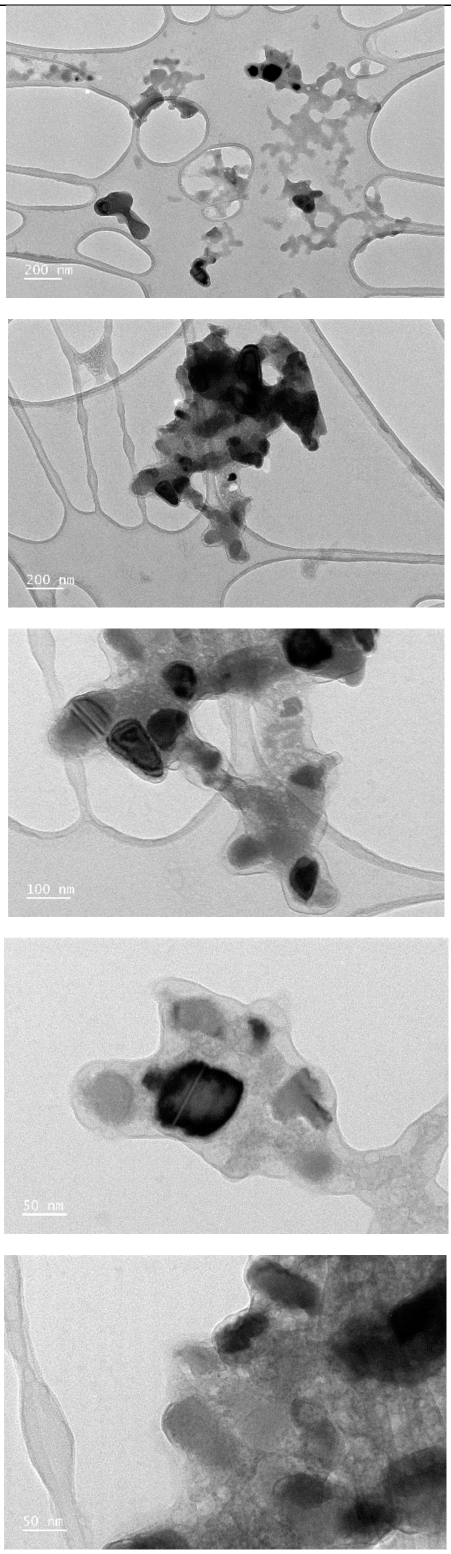

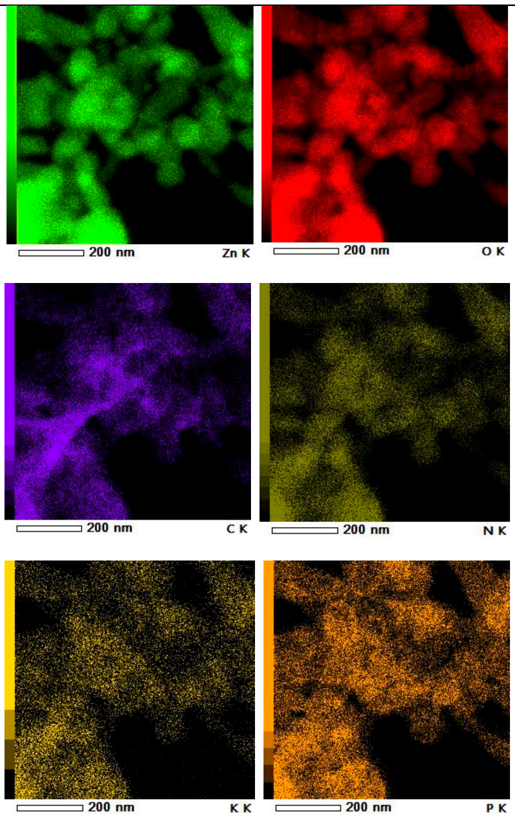
200 nm
Zn K
200 nm
O K
200 nm
C K
200 nm
N K
200 nm
K K
200 nm
P K

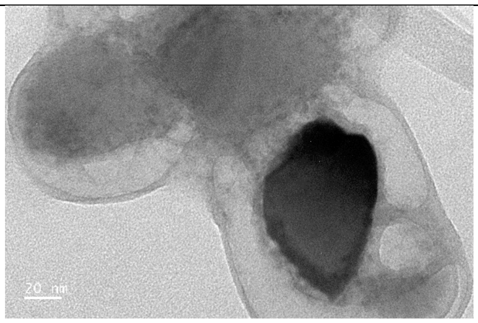
20 nm

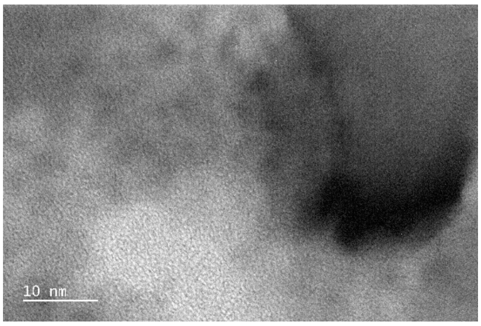
10 nm

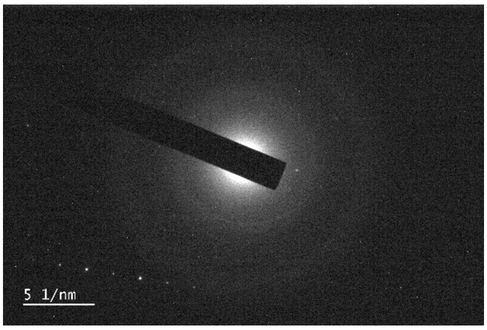
5 1/nm

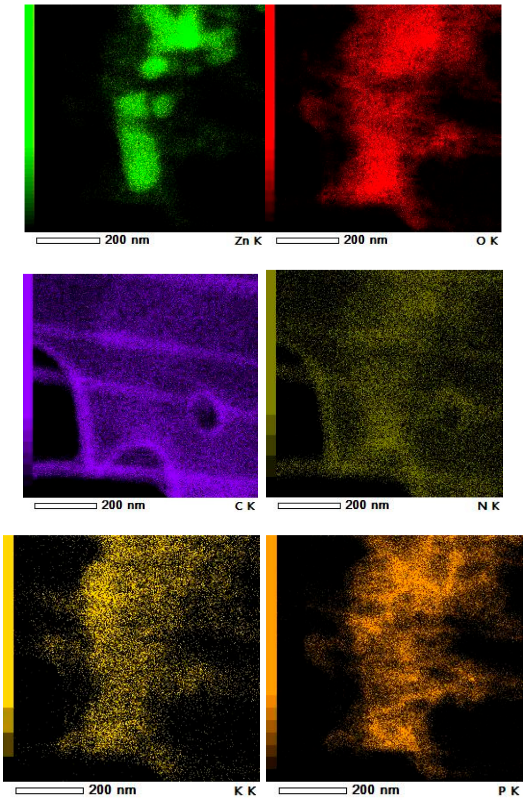
200 nm
Zn K
200 nm
O K
200 nm
C K
200 nm
N K
200 nm
K K
200 nm
P K
